# Material and Technological Advancements in the Recontouring of Maxillofacial, Somatic, and Dental Structures and Their Implications for Auricular Prosthesis: A Case Report

**DOI:** 10.7759/cureus.87206

**Published:** 2025-07-03

**Authors:** Arunoday Kumar, Thingujam Debica, Shamurailatpam Priyadarshini, Rajesh Nongthombam, Manjula Das, Bankim Ningthoujam

**Affiliations:** 1 Department of Prosthodontics and Crown and Bridge, Dental College, Regional Institute of Medical Sciences (RIMS), Imphal, IND; 2 Department of Conservative Dentistry and Endodontics, Dental College, Regional Institute of Medical Sciences (RIMS), Imphal, IND; 3 Department of Prosthodontics, Government Dental College, Silchar, Silchar, IND; 4 Department of Oral and Maxillofacial Surgery, Dental College, Regional Institute of Medical Sciences (RIMS), Imphal, IND

**Keywords:** aesthetics prosthodontics, auricular prosthesis, computer-aided design and computer-aided manufacturing (cad/cam), cosmetic recontouring, dental prosthesis, maxillofacial, polyether ether ketone (peek), poly methyl methacrylate (pmma), room temperature vulcanized (rtv) silicone, somatic

## Abstract

The recontouring of disfigured maxillofacial and dental parts in prosthodontics is a process that goes beyond physical alteration, significantly enhancing patients' psychological well-being. This paper comprehensively analyzes the techniques, materials, and clinical approaches used in the cosmetic recontouring of disfigured parts, such as teeth, gingival tissues, maxillofacial, and somatic (missing body parts like fingers, hands, or prosthetic legs) prostheses. It elaborates on the clinical and laboratory steps involved in the fabrication of auricular prostheses using room temperature vulcanizing (RTV) silicone as a maxillofacial material, which gives a life-like appearance. It helps regain the overall aesthetics of the patient. The prosthesis was well retained through the use of silicone adhesives and the available undercut present at the ear defect site. The prognosis was good in terms of retention, stability, support of the prosthesis, and comfort for the patient, even after six months of follow-up.

Additionally, it gives insights into higher-quality, affordable, and readily available advanced recontour prosthesis materials and modern techniques employed. Recontouring of maxillofacial, somatic, and dental structures is not just a professional duty but a moral imperative, ensuring that the patient's needs and well-being are at the forefront of every decision.

## Introduction

Physical disfigurement, whether due to congenital anomalies, trauma, or surgical excision due to cancer, remains a profound challenge affecting millions of individuals globally. Prosthetic devices have played a pivotal role in mitigating these challenges. Prosthodontics, a dental specialty that focuses on restoring and replacing lost teeth and associated structures - and also cosmetic recontouring in terms of enameloplasty or odontoplasty, laminates, or veneers, which is a conservative prosthodontic rehabilitation procedure to improve the appearance and function of teeth - is at the forefront of this effort. However, a prosthodontist also has expertise in designing and reshaping malformed or deformed maxillofacial structures and other somatic parts, like fingers, hands, or legs, to improve aesthetics and partial function to a great extent [1].

Due to the disfigurement of particular body parts, there is a significant psychological impact on individuals, affecting their self-esteem, social interactions, and overall well-being. The primary objective of recontouring is to restore the natural appearance - such as size, shape, color, and symmetry - thereby improving the patient’s quality of life [2]. While aesthetics is a primary concern, maintaining or improving functionality is equally important.

Cosmetic recontouring is a multidisciplinary approach and can be achieved by sharing expertise and working closely with endodontists, periodontists, and maxillofacial surgeons [3]. This collaboration is essential for managing significant soft tissue disfigurement cases and ensuring that the hard and soft tissues are comprehensively addressed, leading to better aesthetic and functional outcomes [4].

There is a hairline difference between aesthetics and cosmetic prosthodontics. Cosmetic recontouring in prosthodontics enhances visual appearance through treatments like laminates and veneers, crowns, and bridges. In contrast, aesthetic prosthodontics addresses more complex issues - such as missing teeth or parts of the jaw - aiming to restore both function and appearance while achieving overall harmony and balance [5].

Understanding the psychological aspects of individuals is crucial for designing prostheses and managing patients' expectations. Similarly, maxillofacial recontouring can be defined as an approach that involves reshaping disfigured intraoral and extraoral maxillofacial parts using prostheses [6].

Therefore, reviewing different articles and case studies, and highlighting the successes and challenges of aesthetics and cosmetic recontouring of disfigured parts, is crucial, as it offers insight into complex cases and innovative approaches to achieve satisfactory results. 

In recent years, significant progress has been made in the designing and fabrication of prostheses, driven by ongoing research in both academic and industrial sectors. This progress has led to the creation of higher-quality, more affordable prosthetic solutions. Integrating advanced technologies, such as three-dimensional (3D) imaging, modeling, and printing, is particularly noteworthy. These technologies are transforming the traditional manual processes of prosthetic fabrication - often labor-intensive and costly - and enabling the production of customized, patient-specific prostheses with greater precision, efficiency, and cost-effectiveness.

This paper discusses the past and current techniques, materials, clinical outcomes, and future trends in this evolving field, as well as its applied aspects in the recontouring of auricular prostheses, presented in the form of a case report.

## Case presentation

**Figure 1:**
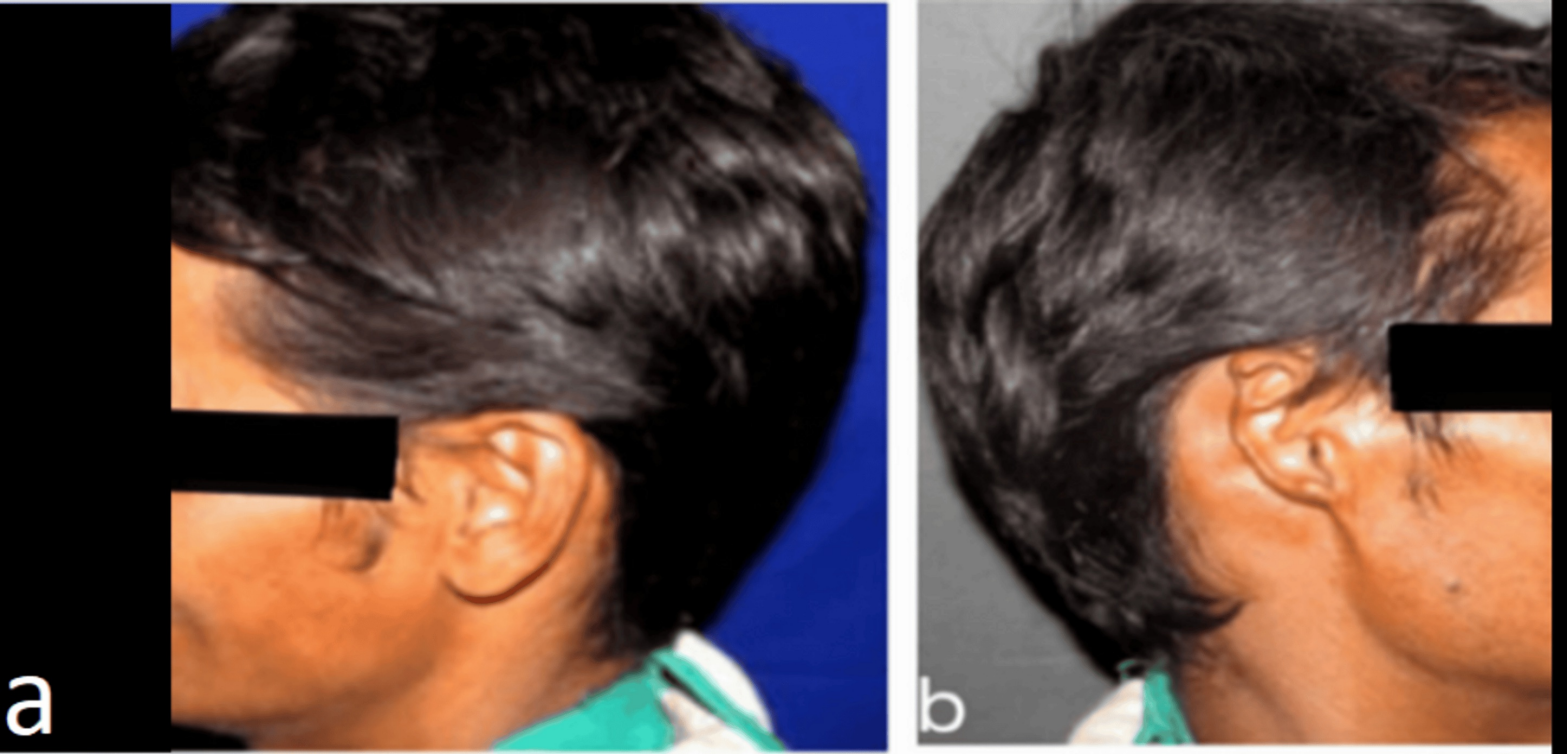
Preoperative view: a) Left lateral and b) Right lateral.

A 36-year-old male patient reported to the Department of Prosthodontics and Crown and Bridge with the chief complaint of the loss of his right ear due to a motorbike accident, and the healing period was uneventful. His primary concern was aesthetics, as shown in Figures 1a-1b.

He did not give any history of cosmetic procedures for the recontouring of the ear defect done in the past, and it was his first visit. No other relevant medical history was reported. The patient was psychologically unhappy because the defect had hampered his overall facial aesthetics. He was philosophical (mentally well adjusted, cooperative, and confident) in nature and was willing to accept treatment that would restore his overall facial aesthetics. The patient was not willing to undergo reconstructive surgery (an alternative treatment option) as it was invasive, costly, and he wanted to avoid surgery. Therefore, a silicone auricular prosthesis was planned for the patient, as this material gives a life-like appearance, though it is artificial. The steps involved in its fabrication are non-surgical and economical for him. Informed consent for the necessary treatment with the silicone auricular prosthesis, as well as permission to use his facial photographs for publication, was obtained from the patient.

**Figure 2:**
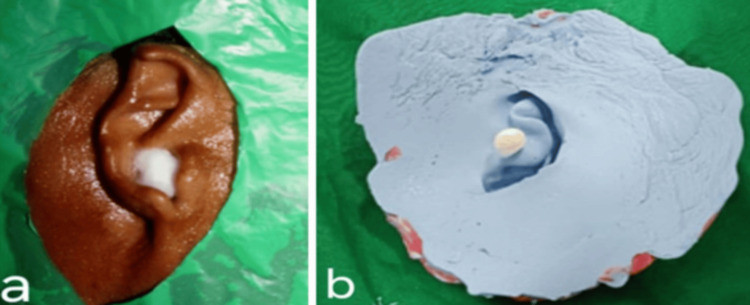
a) Isolation of the ear defect with a polyethylene sheet and b) Impression of the ear defect with alginate impression material.

Clinical and laboratory steps for its fabrication are followed as described below. Firstly, after the application of petroleum jelly (Vaseline Original Skin Protectant Petroleum Jelly, All Skin Types; Unilever, London, United Kingdom), isolating the area with a polyethylene sheet, and placing a cotton bud in the ear canal (Figure 2a), an impression of the traumatized ear or ear defect was made using a customized tray and alginate (Zelgan; Dentsply, Charlotte, NC, USA) impression material, as shown in Figure 2b.

**Figure 3:**
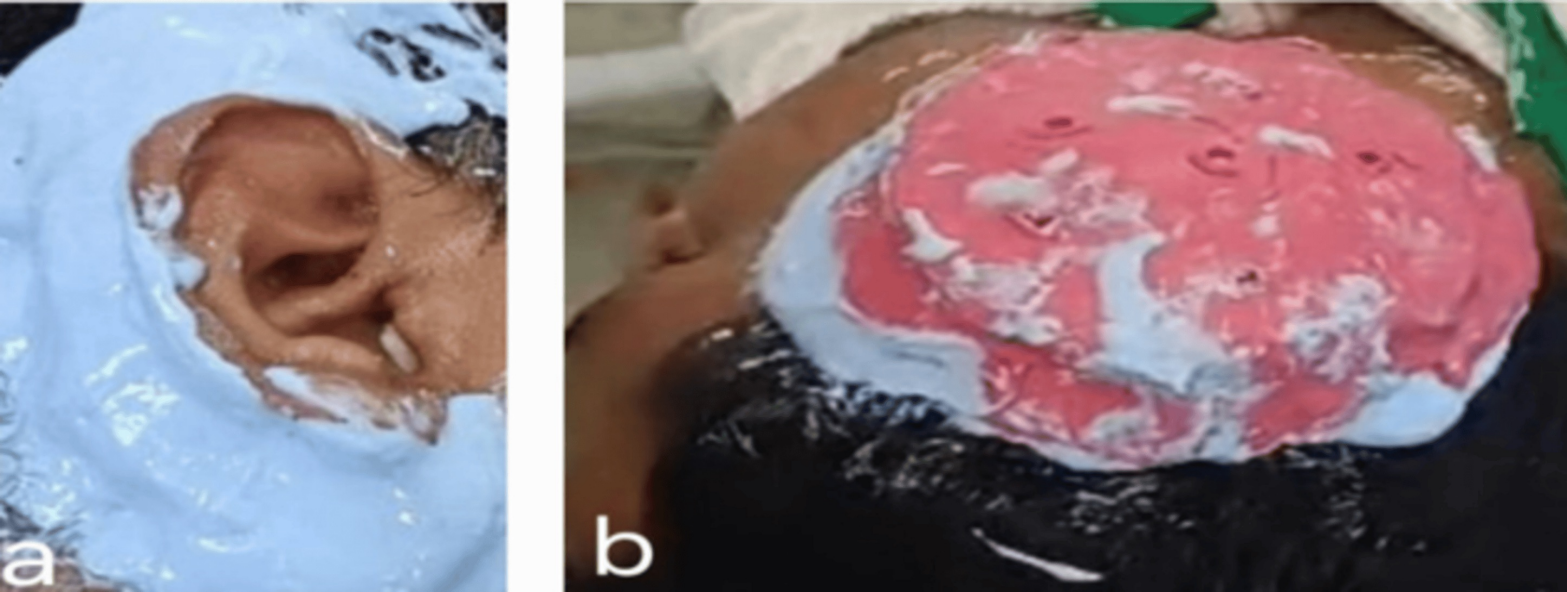
a) First impression taken below the pinna of the ear for support and b) Second impression of the right ear from a donor that closely mimics that of the patient.

**Figure 4:**
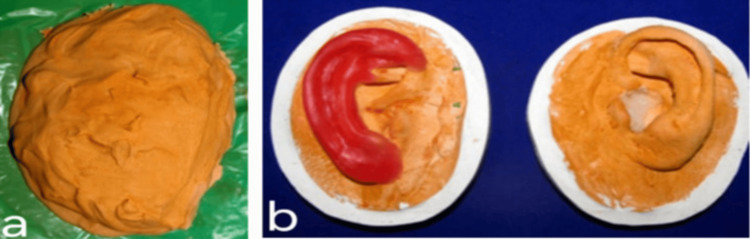
a) Impression poured with die stone and b) Ear pattern carved, adapted to the cast, and compared for symmetrical size and orientation.

**Figure 5:**
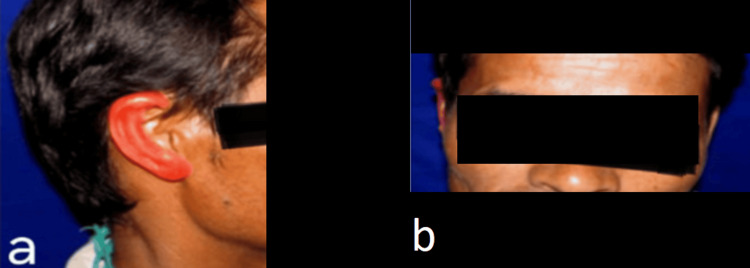
Wax pattern trial for fit, orientation, and aesthetics in the psychological rest position: a) Lateral view and b) Frontal view.

Impression for the normal left ear was also made by following a two-step technique. Firstly, a thin mix of alginate was poured below the pinna of the normal ear to support it from getting flexed, after which the impression of the left ear was made from the top and as a whole, as shown in Figures 3a-3b. This maintained the orientation of the auricular pinna in a 3D plane. Impressions for the right ear defect and the left normal ear were poured with die stone (Figures 4a-4b). From the cast thus obtained, the ear pattern was carved for the right ear defect using the “donor technique,” in which a relative or a person with ear contours that closely mimic those of the patient acts as the donor to make an ear impression. The prepared wax pattern was then adapted to the stone cast symmetrically and was of the same size and orientation when compared with the normal left ear (Figure 4b). Wax pattern trial was done on the patient to verify its fit, symmetrical orientation, and aesthetics in lateral and frontal views (Figures 5a-5b).

**Figure 6:**
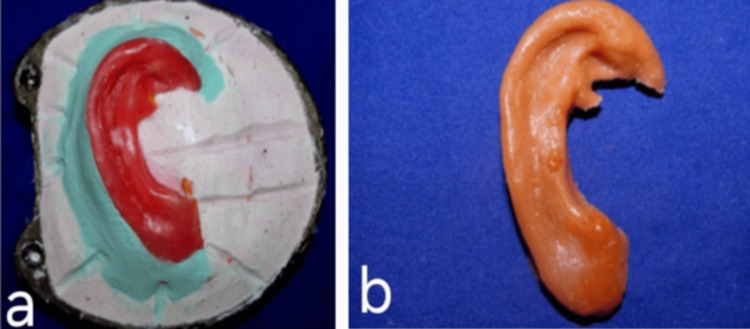
a) Ear pattern was flasked and processed and b) Final processed silicone auricular prosthesis.

The waxed ear was processed with silicone material (room temperature vulcanizing (RTV) silicone, Siloczest Skin Soft), and intrinsic and extrinsic colors were added to match the adjacent normal skin color of the patient, in order to achieve the final aesthetic silicone auricular prosthesis (Figures 6a-6b), which closely mimicked the normal ear contour and skin color of the patient. Visual color matching was done under natural daylight with the patient’s skin. The secondary color was obtained by proportioning and mixing the primary intrinsic and extrinsic colors available to best match the nearby skin color of the patient.

**Figure 7:**
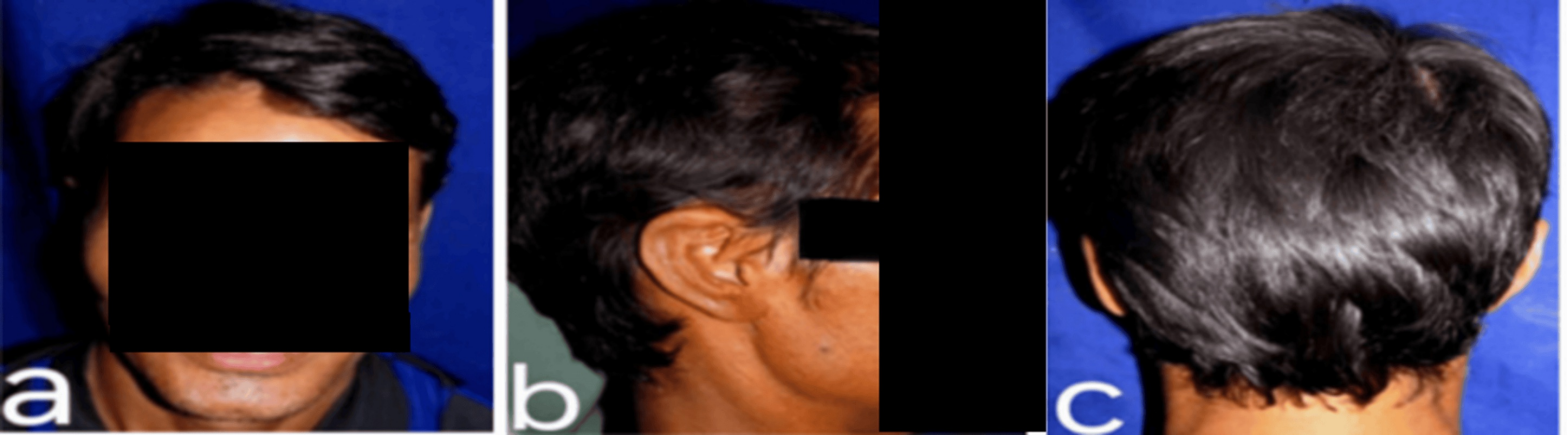
Final auricular prosthesis cemented with silicone adhesive: (a) Lateral view; (b) Frontal view; (c) Dorsal view.

The auricular prosthesis was cemented with silicone adhesives. Retention was provided by the use of silicone adhesives, which are available in the form of paste, liquid, sprays, emulsions, or double-sided tapes. In this case, the prosthesis was well retained because of the silicone adhesives used and the available undercut present in the ear defect. Aesthetics were reverified for symmetrical orientation from the lateral, frontal, and dorsal views of the patient (Figures 7a-7c). The patient was highly satisfied with the prosthesis. He had a good prognosis in terms of retention, stability, and support of the prosthesis, with better comfort and overall psychological and physical health, even after a six-month recall visit.

## Discussion

The articles on maxillofacial and dental recontouring published between 2002 and 2024 were gathered from online databases, including PubMed, DOAJ, ScienceDirect, and Google Scholar. A total of 27 publications from a range of nationally and internationally indexed journals were reviewed to provide the most current information and technological advancements in dental and maxillofacial recontouring. 

In prosthodontics, recontouring involves the adjustment of prosthetic restorations, such as minor modifications (enameloplasty or odontoplasty) to crowns, bridges, and veneers, to improve their fit and restore disfigured parts for function or appearance. Maxillofacial and dental recontouring in prosthodontics offers distinct advantages, as it often requires minimal or no additional surgery and typically provides more aesthetically pleasing results with less invasiveness than plastic surgery [6]. The main goals of maxillofacial prosthetics and rehabilitation are to create a prosthesis that restores the defect, improves function, enhances appearance, and helps the patient reintegrate into society. This approach boosts the patient's morale and contributes to their overall quality of life. The various aspects of recontouring in prosthodontics are pointed out as follows: tooth recontouring, soft tissue recontouring, and maxillofacial recontouring.

Tooth recontouring

Tooth recontouring involves reshaping the enamel to correct minor disfigurements, such as chipped, uneven, or discolored teeth. This procedure is typically performed using fine diamond burs and polishing discs. The amount of enamel removed is minimal, making the procedure conservative and often not requiring anesthesia. In more severe cases, additional restorative techniques, such as bonding or the application of veneers, may be necessary. The selection of technique depends on the extent of the disfigurement and the desired aesthetic outcome. Post-recontouring, the teeth are polished to ensure a smooth surface, reducing the risk of plaque accumulation and enhancing aesthetics [2].

Soft tissue recontouring

Soft tissue recontouring focuses on the gingival tissues, which can become disfigured due to periodontal disease, trauma, or congenital issues. Techniques such as gingivectomy, gingivoplasty, and laser technology are commonly employed to reshape the gum line, enhance aesthetics, and support the underlying dental restorations [2].

Maxillofacial recontouring

For patients with facial disfigurements, prosthetic recontouring is vital in restoring appearance. This involves customizing prosthetic appliances, such as maxillofacial prostheses, to match the patient’s anatomy and skin tone. Advances in materials and digital technology have significantly improved the realism and functionality of these prostheses [2].

Materials used for prosthetic appliances

The materials used in the fabrication of prosthetic appliances in prosthodontics have evolved significantly from the early stages to the present, reflecting advancements in both material science and fabrication techniques. Initially, prosthetic devices were crafted from rudimentary materials such as ivory, wood, and leather cups for lost limbs and legs in war [7]. The introduction of vulcanized rubber in the mid-19th century marked a significant improvement, providing a more flexible and resilient option for dentures and other prosthetic devices [8]. As material science progressed, acrylic resins emerged in the 20th century as a revolutionary development due to their superior aesthetics, ease of manipulation, and biocompatibility. Concurrently, metal alloys such as gold, cobalt-chromium, and titanium were incorporated for their strength and stability, particularly in load-bearing prostheses like dental implants, and maxillary and mandibular frameworks in resection cases [9].

In recent decades, the development of silicone elastomers and advanced composite materials has further refined the field, allowing for highly customizable prosthetic devices that closely mimic the appearance and function of natural tissues. These modern materials are designed to be biocompatible, durable, and capable of withstanding the mechanical and thermal stresses of the oral environment, representing the culmination of centuries of innovation in prosthodontic materials. The materials used for recontouring vary depending on whether the procedure is performed on natural teeth, prosthetic restorations, or natural living tissues. For natural teeth, recontouring of enamel and dentin is the primary tissue involved, whereas for prosthetic restorations, materials such as porcelain, composite resin, zirconia, silicone, etc., may be reshaped or recontoured to fit into the natural tissues for enhanced aesthetics. The selection of materials plays a significant role in the ease of recontouring and the quality of the outcome. Table 1 provides a comprehensive overview of the evolution of prosthetic materials in prosthodontics, including their characteristics and uses.

**Table 1:** Evolution of materials used for prosthetic devices over time. CAD/CAM, Computer-aided design and computer-aided manufacturing; PEEK, Polyether ether ketone; PMMA, Polymethyl methacrylate

Time	Material	Characteristics	Usage in prosthodontics & advantage
Ancient Times [2,7,10]	Ivory, wood, leather cups	Natural materials, limited durability, and aesthetic qualities	Early dentures, lost limbs, and leg prostheses were readily available, accessible to shape
19th Century [7,9,10]	Vulcanized rubber	Flexible, resilient, better fit, and comfort compared to earlier materials	Dentures, essential prosthetic appliances, improved fit, more comfortable, cost-effective
	Porcelain	Hard, brittle, aesthetic, resembles natural teeth	Denture teeth and crowns, highly aesthetic, resemble natural teeth
	Gold alloys	High-strength, corrosion-resistant, biocompatible	Crowns, bridges, inlays, onlays, durable, biocompatible, excellent fit
20th Century [4,7-10]	Acrylic resin	Aesthetic, easy to manipulate, lightweight, cost-effective	Dentures, denture bases, temporary crowns, highly aesthetic, easy to work with, lightweight
	Cobalt-chromium alloys	High-strength, corrosion-resistant, lightweight	Partial denture framework crowns, durable, lightweight, and have good corrosion resistance
	Stainless steel	Durable, cost-effective, corrosion-resistant	Temporary crowns, orthodontic appliances, strong, affordable, widely available
	Aluminum-titanium alloys	Biocompatible, strong, lightweight, corrosion-resistant	Dental implants, prosthetic frameworks, highly biocompatible, strong yet lightweight, excellent corrosion resistance
	Composite resins	Aesthetic, tooth-colored, good wear resistance	Fillings, veneers, crowns, tooth-colored, versatile, less invasive
	Silicone elastomers	Flexible, biocompatible, used for soft tissue replacement	Maxillofacial prostheses, facial prosthetics, flexible, biocompatible, suitable for soft tissue replication
	Zirconia	High strength, aesthetic, tooth-colored, biocompatible	Crowns, bridges, implant abutments, highly aesthetic, very strong, biocompatible
	Carbon fiber composites	Lightweight, high strength-to-weight ratio	Used in prosthetic limbs and sockets, durable, strong, reduces overall weight
	Silicone elastomers	Flexible, skin-friendly, durable	Used in liners, interfaces, and sockets, comfortable, reduces skin irritation, long-lasting
Present Century (2000 to 2024) [4,7,8,10]	Advanced carbon fiber and fiberglass	Enhanced strength and flexibility	Used in high-performance prosthetic limbs, lightweight, strong, customizable
	Polyurethane and thermoplastic materials	Flexible, mouldable, durable	Used in sockets, liners, and prosthetic components, good fit, adaptable, comfortable
	CAD/CAM ceramics	Precision, high aesthetic value, customizable	Crowns, bridges, inlays, onlays, highly aesthetic, precise fit, durable
	PEEK	High strength, biocompatible, lightweight, aesthetic	Implant frameworks, removable prostheses, lightweight, biocompatible, good mechanical properties
	PMMA	Transparent, durable, light-weight, thermoplastic, good impact strength, and biocompatible	Used in the fabrication of artificial teeth, temporary and permanent denture bases, prosthetic limbs, fingers, ocular prosthesis

The materials used for different prostheses and their clinical outcome are described in Table 2.

**Table 2:** Dental materials used for recontouring. PMMA, Polymethyl methacrylate

Type of prosthesis for recontouring disfigured parts		Material used	Clinical outcome
Maxillofacial and somatic prostheses [1,3-5,10]	Ocular prostheses	Silicone	Artificial eyes, flexible, biocompatible, natural appearance
		PMMA	Artificial eyes, rigid, clear, durable
	Nasal prostheses	Silicone	Flexible, skin-friendly, customizable, custom-made artificial noses
		Acrylic	Rigid, moderate weight, less flexible, artificial nose
	Auricular	Silicone	Flexible, lightweight, skin-friendly, artificial ears
		Acrylic	Rigid, moderate-weight, artificial ears
	Maxillary resection prostheses	PMMA	Upper jaw reconstruction, rigid, durable, mouldable
		Titanium	Maxillary implants, strong, biocompatible, durable
	Mandibular resection prostheses	Titanium	Mandibular implants, rigid, durable
		PMMA	Lower jaw reconstruction, strong, biocompatible, durable
	Arm, leg Prostheses	Carbon fiber	Arm, leg replacements, enhancements, lightweight, strong, durable
		Titanium	Strong, biocompatible, lightweight
Dental prostheses [2,4,5,7-10]	Crowns	Ceramic	Tooth restoration, hard, durable, natural appearance
		Porcelain	Aesthetic, durable dental crowns
		Zirconia	Strong, biocompatible, natural appearance, crowns, bridges
	Bridges	Ceramic	Aesthetic, durable, tooth replacement
		Metal	Strong, durable, often used with ceramics, dental bridges
	Dentures	Acrylic	Lightweight, adjustable, cost-effective, removable replacement for missing teeth
		Nylon	Flexible, durable, flexible dentures
	Implants	Titanium	Strong, biocompatible, durable, permanent teeth replacement
		Zirconia	Strong, biocompatible, dental implants
	Veneers	Porcelain	Thin, aesthetic, natural look, tooth appearance enhancement
		Composite	Less durable, cost-effective, dental veneers

Soft tissue prosthetic materials are used for the recontouring of living tissues and maxillofacial structures. Soft tissue materials, like collagen membranes, allografts, xenografts, and synthetic grafts, are employed in gum grafting and periodontal regeneration procedures. These offer scaffolding for new tissue growth and aid healing. Hyaluronic acid gels enhance soft tissue regeneration, while silicone elastomers are used in maxillofacial prosthetics to replicate the look and feel of soft tissues [10]. Prosthetic materials used for dental recontouring include acrylic resin, porcelain, ceramics, zirconia, titanium, and cobalt-chromium alloys, which create durable and aesthetically pleasing dental devices like dentures, crowns, bridges, and implants. Acrylic resin is commonly used for dentures due to its ease of manipulation and cost-effectiveness, while porcelain and zirconia provide excellent aesthetics in crowns and bridges. Titanium's biocompatibility makes it ideal for dental implants, and cobalt-chromium alloys are valued for their strength in partial denture frameworks [11]. Soft liners, made from silicone or modified acrylic, are applied to dentures for patient comfort. Advances in these materials, including the development of bioactive and biomimetic options, continue to improve the outcomes of dental treatments, enhancing both functionality and aesthetics for patients. Commonly used restorative materials include amalgam, a durable metal mix, and composite resins, which are tooth-colored plastics. Glass ionomer cement releases fluoride, and ceramics are valued for their aesthetics and utility in restorations. Impression materials, like alginate and polyvinyl siloxane, are essential for accurate dental molds. Advances in dental materials continue to improve dental care's durability, aesthetics, and functionality. The choice of material is determined by factors such as the tooth's location, the extent of the recontouring required, and the patient’s aesthetic expectations.

Advances in the fabrication approach of cosmetic prosthesis

The fabrication of cosmetic prostheses has undergone a notable transformation from traditional to modern approaches, reflecting the technological and methodological advancements of its time. Traditionally, the process was manual and artisanal, relying on techniques such as sculpting and molding with materials like plaster and wax. Prosthetists would create custom molds directly from the patient's anatomy, followed by meticulous handcrafting of the prosthesis using materials like silicone or polyurethane [12]. This traditional method, though personalized, often faced challenges related to the time-consuming nature of production and the limitations of manual precision [13]. In contrast, modern fabrication approaches leverage technological innovations and are used to enhance efficiency and accuracy. Integrating computer-aided design (CAD) and 3D printing has revolutionized the field, allowing for highly precise and customized prostheses with reduced turnaround times. With CAD, practitioners can create detailed digital prosthesis models, which can be directly translated into physical forms using 3D printing technology [13]. This modern approach streamlines the production process and uses advanced materials such as high-performance silicones and lightweight composites that offer improved durability and a more natural appearance. Modern techniques often incorporate advanced imaging technologies, like computed tomography (CT) scans, to ensure a perfect fit and better aesthetic outcomes. The shift from traditional to modern methods underscores a significant leap in the field of cosmetic prosthetics, enhancing both the functionality and the visual appeal of prosthetic devices. Different fabrication approaches for various prosthetic appliances, in terms of material selection and their respective merits and demerits, are compared in Table 3.

**Table 3:** Detailed prosthesis types and applications with merits and demerits.

Prosthesis type: application	Approach: material used	Merits	Demerits
Finger: Restoration of function and appearance of lost fingers [14,15]	Traditional: Silicone, polyurethane	Highly customizable, good aesthetic results	Time-consuming, less precise, frequent adjustments needed
	Modern: Plastics, lightweight composites, silicones	Accurate fit, enhanced functionality, quick process	High cost, requires advanced technology, may need calibration
Auricular: Reconstructs and restores the appearance of a lost natural ear [16-18]	Traditional: Silicone, latex	Personalized fit, natural look	Less precise, labor-intensive
	Modern: Advanced silicones, medical-grade polymers	Precise fit improved durability	Initial costs can be high, technology-dependent, and may require fitting adjustments
Nasal: Reconstructs and restores the appearance of a lost natural nose [19-21]	Traditional: Silicone, latex	Custom-fit, aesthetically pleasing	Manual fitting may be imprecise, less durable
	Modern: Advanced silicones, medical-grade polymers	Precise fit improved durability, better appearance	High cost, technology requirement, may need periodic updates
Ocular: Reconstructs and restores the appearance of the lost eye [22-24]	Traditional: Glass, acrylic	Natural look achievable, customizable	Less comfortable, less precise fitting
	Modern: Acrylic specialized polymers	Highly accurate, mimics natural eye movement, comfortable	Expensive, requires high-tech equipment, may be less flexible
Smile design: Enhances the beauty of the smile [25-27]	Traditional: Porcelain, composite resins	Customizable; improved aesthetics	Labor-intensive, less precise
	Modern: Ceramic advanced composites	Precise fit, better aesthetics, efficient production	Costly, requires specialized technology, and may need adjustments over time

The fabrication of an auricular prosthesis and its outcome depend on the skill of the prosthodontist - specifically, how precisely and with what expertise the prosthesis is made. A pilot study by Mohamed et al. (2013) concluded that the triple-layer technique followed for ear impressions gives more accurate models than the conventional impression technique when compared with patients' actual dimensions [17]. A clinical report by Yadav et al. (2017) describes the use of CT scanning, CAD, and rapid prototyping to fabricate an auricular prosthesis with high precision [18]. A case series by Leonardi et al. (2008) reported on 21 titanium implants supporting auricular prostheses, placed due to excessive auricular loss, and concluded that the use of implants not only supports the prosthesis but also increases its retention [24].

This article focuses on a non-surgical, cost-effective procedure involving the fabrication of an auricular prosthesis with good retention, stability, support, and improved patient comfort, along with enhanced aesthetics. Although fabricating an aesthetic auricular prosthesis requires thorough training, the final design allows patients with ear defects to use it without encountering accessibility barriers.

## Conclusions

Cosmetic recontouring of disfigured parts in prosthodontics is a complex and multifaceted field, requiring a thorough understanding of aesthetic and functional principles. Integrating modern technologies, advanced materials, and a multidisciplinary approach is essential for achieving optimal results. As the field continues to evolve, the potential for improving patient outcomes and quality of life will expand, making cosmetic recontouring an indispensable aspect of prosthodontic care. Advances in digital technology have further refined the process, making it more predictable and patient-friendly. With proper planning and execution, cosmetic recontouring can provide lasting results and high patient satisfaction.
